# Hysteresis in the underdamped three-layer model

**DOI:** 10.1038/s41598-020-67779-9

**Published:** 2020-07-02

**Authors:** Li-Ping Jia, Jasmina Tekić

**Affiliations:** 1 Department of Physics, Longdong University, Qingyang, 745000 China; 2 Department of Theoretical and Condensed Matter Physics-020, “Vinča” Institute of Nuclear Sciences, National Institute of the Republic of Serbia, University of Belgrade, PO Box 522, 11001 Belgrade, Serbia

**Keywords:** Mathematics and computing, Physics

## Abstract

The hysteretic phenomena were investigated in the three-layer model consisting of a chain of harmonically interacting atoms confined between two rigid periodical substrate potentials, where the top substrate was driven by a external force. The pinning to running and the running-to pinning transitions were examined as the driving force was varied and the influence of the equilibrium spacing and strength of the interaction of the particles in the middle layer on the static and kinetic friction force analyzed in detail. The parameter space in which the friction forces could reach their maxima or minima was determined. These results could be interesting for the selection of lubricant materials and minimization of energy loss in tribology.

## Introduction

More than 70 years ago, the dislocation dynamics in crystals was first described by Dehlinger with the classical one dimensional (1D) Frenkel–Kontorova (FK) model^1^. Later, the FK model has been applied extensively in many problems of nonlinear physics, including: vortex lattice in Superconductivity^2^, Josephson junctions systems^3^, charge or spin density waves^4,5^, tribology^6–9^, etc. Especially in connection with solid friction phenomena, the application of driven underdamped Frenkel–Kontorova-type model has received an increasing attention as a possible interpretative tool that provides a deep understanding of the complex field of nanotribology^10–13^.

In the recent experimental studies, the influence of the chain stiffness on the kinetic friction force was investigated in three-layer model with incommensurate structure. According to these results, for larger chain stiffness, golden mean incommensurate structure presents a very regular periodic motion. Under the same chain stiffness, the golden mean structure has higher kinetic friction than spiral mean structure^13^. These results are useful for understanding the energy loss in nanotribology. In Ref.^14^, hysteresis in the two-dimensional Frenkel–Kontorova model was studied where the dependence of four critical forces on interatomic strength, winding number, misfit angle, and external driving force was examined^14^ . In Ref.^15^, hysteresis was found even in overdamped one-dimensional Frenkel–Kontorova model. Hysteresis and the friction phenomena have been studied previously in two layer models^14–16^. However, hysteresis in the underdamped three-layer model is seldom studied though in real situations, the so-called "third bodies" are often mediated between two solids, which act like a lubricant film.

In the present paper, we consider a three layer model, which consist of a driven underdamped chain of anharmonically interacting atoms confined between two periodic sinusoidal substrate potentials. Starting from the three-layer model proposed by Bruan et al. in Ref.^13^, where a chain of interacting particles is embedded between two rigid sinusoidal substrates and the top substrate is pulled through a spring connected to a stage with uniform motion, we in a way improve the model by changing the spring for a gradually increasing external driving force. We will examine the influence of the chain stiffness and equilibrium spacing for the commensurate structure on the maximum static and the maximum kinetic friction, when the top substrate is driven by the force.

## Results

**Figure 1:**
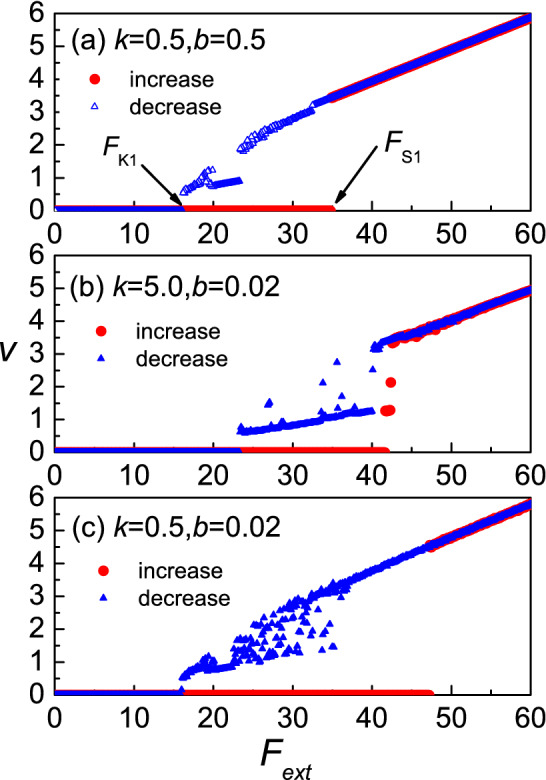
The upper substrate velocity $v$ as a function of the driving force $F_{ext}$ in different equilibrium spacing $b$ and particle interaction strength $k$ regimes:$({\mathbf{a}})\,k = 0.5,\,\,b = 0.5;\,({\mathbf{b}})\,k = 5.0,\,b = 0.02;\,({\mathbf{c}})\,k = 0.5,b = 0.02.$ $F_{ext}$ is the driving force normalized to $F_{ext0} = 1N$ and $v$ is the upper substrate to $v_{0} = \sqrt {{{2\pi } \mathord{\left/ {\vphantom {{2\pi } a}} \right. \kern-\nulldelimiterspace} a}} = 4.0 \times 10^{ - 7} \,{\text{m}}/{\text{s}}$.

**Figure 2:**
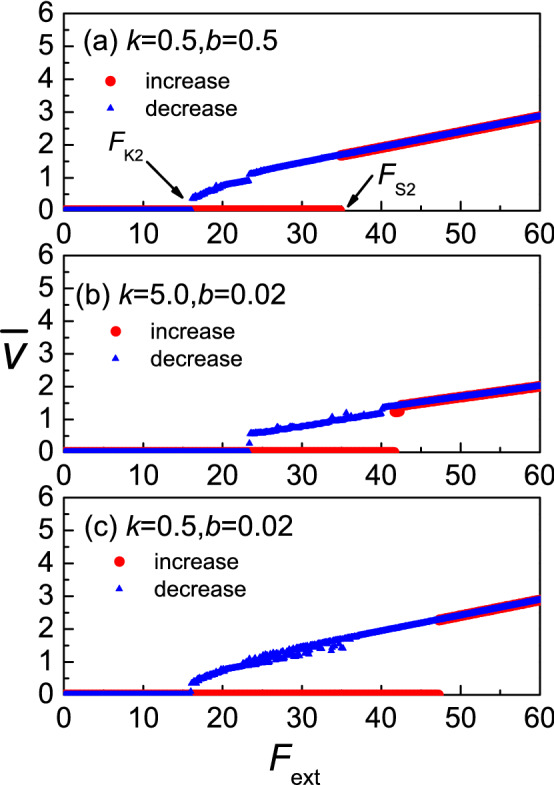
The average velocity of middle layer $\overline{v}$ as a function of the driving force $F_{ext}$ in different equilibrium spacing $b$ and particle interaction strength $k$ regimes:$({\mathbf{a}})\,k = 0.5,\,b = 0.5;({\mathbf{b}})\,k = 5.0,\,b = 0.02;\,({\mathbf{c}})\,k = 0.5,\,b = 0.02.$

In Fig. 1, the variation of the upper substrate velocity $v$ as a function of the driving force $F_{ext}$ in the three different regimes of the equilibrium spacing $b$ and the particle interaction strength $k$ is presented. As the force $F_{ext}$ increases from zero, at some value, the velocity $v$ of the substrate potential of the upper layer is changed from zero to non-zero, we define that point as the maximum static friction $F_{s1}$. With the further increase of driving force the system remains in the running state. If from there we begin to reduce the driving force slowly, at some value of driving force the velocity of the upper layer droops to zero. We define this point as the kinetic friction force $F_{k1}$. A hysteretic phenomenon takes place in this case when $F_{s1} = F_{k1}$ (see Fig. 1). As we can see, both parameters of $k$ and $b$ strongly influence $F_{s1}$ and $F_{k1}$. In the same way as in Fig. 1, we analyze the average velocity of the middle layer $\overline{v}$, wich is presented as a function of the driving force $F_{ext}$ in the three different regimes of $b$ and $k$ in Fig. 2. Again, we define two critical forces as: $F_{s2}$ and $F_{k2}$. The hysteretic phenomenon takes place in this case when $F_{s2} = F_{k2}$ (see Fig. 2). As we can see, for the same $k$ and $b$, $F_{s1} = F_{s2}$, $F_{k1} = F_{k2}$. We will consider the case of the system when $F_{s1} = F_{s2} = F_{s}$, and $F_{k1} = F_{k2} = F_{k}$. Therefore, we will only examine $F_{s}$, and $F_{k}$, which is enough to illustrate the behavior. In order to understand the hysteretic phenomenon in more detail we will examine how the system parameters affect the static and kinetic friction forces.

**Figure 3:**
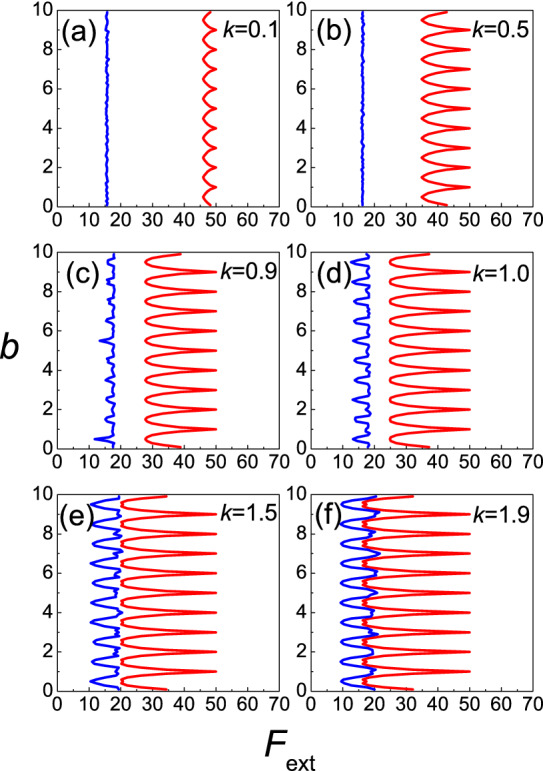
Contour plot of the static friction $F_{s}$ and the kinetic force $F_{k}$ as a function of equilibrium spacing $b$ with different strength $k$: $({\mathbf{a}})\,k = 0.1;\,({\mathbf{b}})\,k = 0.5;\,({\mathbf{c}})\,k = 0.9;\,({\mathbf{d}})\,k = 1.0;\,({\mathbf{e}})\,k = 1.5;\,({\mathbf{f}})\,k = 1.9.$ (the red line represents the static friction and the blue line represents the kinetic friction.).

The dependence of the static friction $F_{s}$ and the kinetic friction $F_{k}$ on the external driving force $F_{ext}$ and equilibrium spacing $b$ for different strength $k$ is given in Fig. 3a–f (the red line represents the static friction and the blue line represents the kinetic friction). The results show that the difference between the maximum and the minimum values of the both $F_{s}$, and $F_{k}$ increases as the strength $k$ increases. However, while the maxima of $F_{k}$ increase with $k$, the maximum value of the static friction force, on the other hand, remains independent of the parameter $k$ and keeps the constant value $F_{s} = 50$. Meanwhile, if we look at the minima, in both cases for $F_{s}$, and $F_{k}$, their values decrease as the parameter $k$ increases. The dependence of the static friction force on the parameter $b$ is periodic with the periodicity, which is equal 1. Nevertheless, the dependence of the kinetic friction force on the parameter $b$ is quasi-periodic with the periodicity, which is also 1. Thus, we can consider only one period, which would be enough to analyze the behavior.

**Figure 4:**
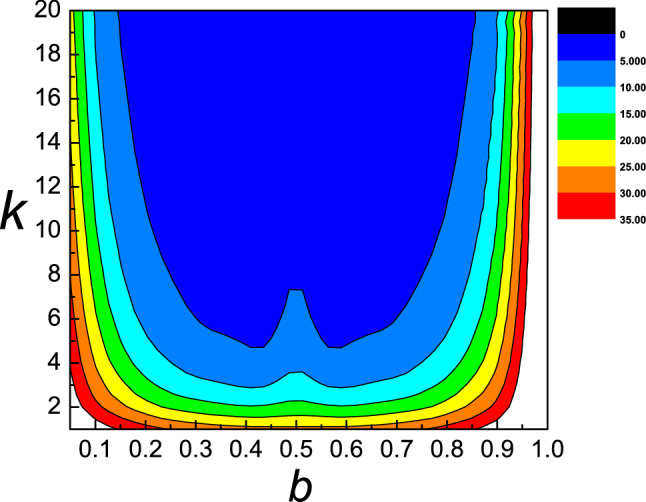
Contour plot of the static friction $F_{s}$ as a function of equilibrium spacing $b$ and different strength $k$. Different colors in the figure represent different values of the static friction.

**Figure 5:**
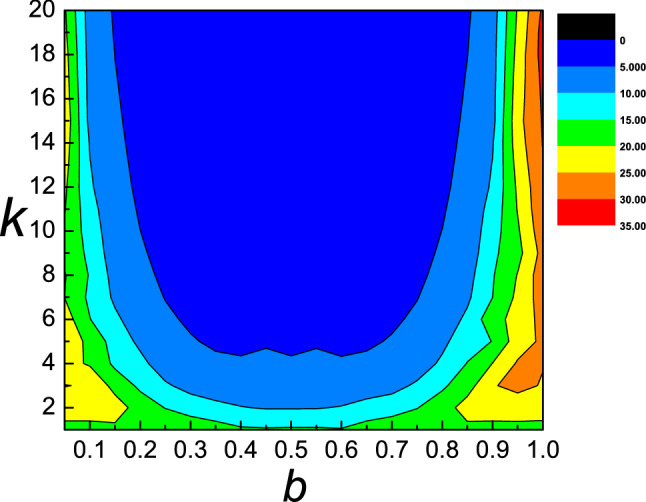
Contour plot of the kinetic friction $F_{k}$ as a function of equilibrium spacing $b$ and different strength $k$. Different colors in the figure represent different values of the kinetic friction.

The dependence of the static friction $F_{s}$ as a function of equilibrium spacing $b$ and different strength $k$ is presented in Fig. 4. We can see that when $b$ is in the range of 0.1–0.4, $F_{s}$ decrease as both the equilibrium spacing $b$ and different strength $k$ increase. When $b$ is in the range of 0.4–0.6, $F_{s}$ decrease as different strength $k$ increase. However, when $b$ is in the range of 0.6–1.0, $F_{s}$ increases with the increase of $b$ and decreases with the increase of $k$. The dependence of the kinetic friction $F_{s}$ as a function of equilibrium spacing $b$ and different strength $k$ is presented in Fig. 5. The system has the least kinetic friction for the values of $k$ and $b$, which corresponds to blue region of the plane, while when $b$ is between 0.9 and 1.0, it has the most kinetic friction.

## Discussion

In the present paper, the dynamics of an underdamped three-layer model, which consists of a harmonic chain confined between two periodical substrate potentials has been investigated. The obtained results showed that when the upper substrate was driven by the external force $F_{ext}$, hysteresis phenomenon would appear not only in the upper substrate but also in the middle chain. The static and kinetic friction forces for both layers have been analyzed, where the particular case when both layers had the same static and the same dynamic friction forces was considered. Their dependence on the equilibrium spacing and the strength of the interparticle interaction in the middle layer was examined in detailed and a diagram, which clearly shows the regions with the maximum or minimum frictions determined. The presented results can provide reference for the selection of lubricant materials and the reduction of the energy loss in the systems.

A similar experiment can be done a graphite flake between the same crystalline surfaces (for example, iron crystal). The external driving force acts on the upper layer, but there is no driving force in the middle layer, the bottom substrate potential is fixed. In this case, the middle layer is lubricant. If the driving force is not large enough, the middle and upper layer remains motionless. If the driving force reaches a certain critical force, the upper and middle layers begin to move, the critical force is the maximum static friction. The maximum kinetic friction force has a similar definition. The parameters of lubricant determine the maximum static friction and the maximum kinetic friction force^13^.

## Methods

**Figure 6:**
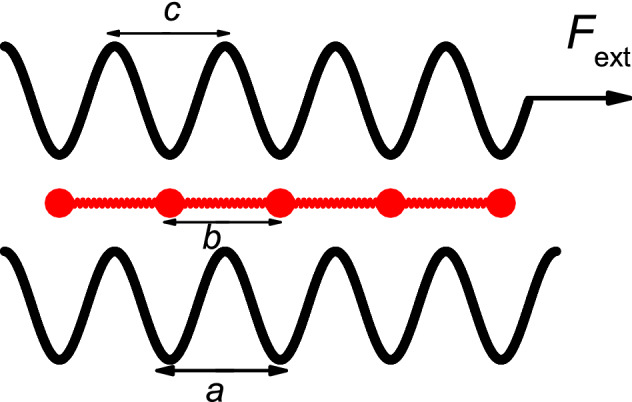
Schematic drawing of the underdamped three-layer model.

The three-layer model consists of a chain of $N$ harmonically interacting particles interposed between two rigid generally sinusoidal substrates as shown in Fig. 6. The top substrate (the upper layer) is driven by the force $F_{ext}$, and it satisfies the following equations of motions^13^:

1 $$M\ddot{X}_{top} = - \sum\limits_{i = 1}^{N} {\gamma (\dot{X}_{top} - \dot{x}_{i} )} + \sum\limits_{i = 1}^{N} \frac{1}{2} \left[ {\sin \frac{{2\pi (X_{top} - x_{i} )}}{c}} \right] + F_{ext} .$$

The equation of motion of the $i$-th particle of the middle layer is given as

2 $$m\ddot{x}_{i} = - \gamma \dot{x}_{i} - \gamma (\dot{x}_{i} - \dot{X}_{top} ) + \frac{d}{{dx_{i} }}\sum\limits_{i \ne j} {V\left( {\left| {x_{i} - x_{j} } \right|} \right)} + \frac{1}{2}\left[ {\sin \frac{{2\pi x_{i} }}{a} + \sin \frac{{2\pi (x_{i} - X_{top} )}}{c}} \right].$$

In the whole process, the lower substrate (the lower layer) has been fixed. In Eqs. (1) and (2), $m$ and $M$ are the mass of the particle of the middle layer and the upper substrate, while $x_{i} (i = 1,2,3 \ldots N)$ and $X_{top}$ stand for their coordinates, respectively. $\gamma$ is a phenomenological parameter substituting for various sources of dissipation, required to achieve a stationary state (here, we choose $\gamma = 0.2$), but otherwise with no major role in the following. $V(X)$ is the interatomic interaction potential between particles in the middle layer. We assume that it has the following harmonic form:

3 $$V(X) = \frac{k}{2}[(X - b)^{2} ],$$

with a strength $k$ and equilibrium spacing $b$. $X$ is the difference of the coordinates between the nearest neighbors. We have used dimensionless units and consider the particles mass $m = M = 1.0$, and the equal periods of all three layers $a = c = 1.0$. The last (sinusoidal) terms in Eqs. (1) and (2) represent the on-site interaction between the particles and the substrates^13^. The sinusoidal terms in Eqs. (1) and (2) represent the on-site interaction between the particles and the substrates. The magnitudes of the rigid potentials are chosen such that the same factor (1/2) sits in front of their derivatives in the equations of motion. The Eqs. (1) and (2) have been integrated using the fourth-order Runge–Kutta method. The time step used in the simulations was $0.02\tau$, and a time interval of $100\tau$ was used as a relaxation time to allow the system to reach the steady state^13^. The force was varied with the step of $10^{ - 4}$.

The bottom substrate potential is fixed, and its damped force and force on the particles in the middle layer can be regarded as friction, which hinders the movement of the particle chain in the middle layer. In the same way, the force and damped force of the particle chain in the middle layer on the top substrate potential can also be regarded as friction, which hinders the movement of the upper layer of the bottom substrate potential, so this model can be regarded as a lubricate friction model.

## Data Availability

The program used in this article can be provided by the corresponding author.
